# A Diverse Ant Fauna from the Mid-Cretaceous of Myanmar (Hymenoptera: Formicidae)

**DOI:** 10.1371/journal.pone.0093627

**Published:** 2014-04-03

**Authors:** Phillip Barden, David Grimaldi

**Affiliations:** 1 Division of Invertebrate Zoology, American Museum of Natural History, New York, New York, United States of America; 2 Richard Gilder Graduate School, American Museum of Natural History, New York, New York, United States of America; Field Museum of Natural History, United States of America

## Abstract

A new collection of 24 wingless ant specimens from mid-Cretaceous Burmese amber (Albian-Cenomanian, 99 Ma) comprises nine new species belonging to the genus *Sphecomyrmodes* Engel and Grimaldi. Described taxa vary considerably with regard to total size, head and body proportion, cuticular sculpturing, and petiole structure while all species are unified by a distinct shared character. The assemblage represents the largest known diversification of closely related Cretaceous ants with respect to species number. These stem-group ants exhibit some characteristics previously known only from their extant counterparts along with presumed plesiomorphic morphology. Consequently, their morphology may inform hypotheses relating to basal relationships and general patterns of ant evolution. These and other uncovered Cretaceous species indicate that stem-group ants are not simply wasp-like, transitional formicids, but rather a group of considerable adaptive diversity, exhibiting innovations analogous to what crown-group ants would echo 100 million years later.

## Introduction

Fossils from the Cretaceous are the only windows into ancient morphotypes exhibited by now extinct stem-group ants. The earliest ants known are Late Albian to Early Cenomanian (∼99–100 mya) in age from approximately contemporaneous Charentese and Burmese amber of France and Myanmar, respectively [1]. Although there are insect yielding fossil deposits older than 100 million years, no definitive ants have been recovered from these localities. Along with the very oldest ant fossils, there are younger Cretaceous deposits that shed light on early ant history: species have been described from New Jersey amber (92 mya), two Siberian amber deposits (∼85 and ∼80 mya), and Canadian amber (∼79 mya) [1]. Most Cretaceous species are not readily assignable to modern subfamilies and are either *incertae sedis* or belong to taxonomic groups erected to accommodate presumed basal lineages. These stem-group taxa persist only into Canadian amber from the Late Cretaceous [2]. The majority of stem-group ant taxa belong to the subfamily Sphecomyrminae, which possess some, but not all, of the synapomorphies associated with living crown-group ants. Namely, while sphecomyrmine ants possess a petiole and metapleural gland opening, the scape is shorter than those found in living ants today [3]. Over the past 20 years, new fossil discoveries have demonstrated that ants within the Sphecomyrminae are surprisingly diverse, exhibiting unusual feeding morphology in particular [4]–[8].

While there are a number of Cretaceous amber deposits, ants from this time period are exceedingly rare and not particularly speciose. In the case of Burmese amber, ants comprise approximately 0.2% of all insect inclusions [9]–[10] with a total of seven described species [11]. This low species number provides little resolution for describing patterns of early ant diversification and makes a significant novel species radiation particularly noteworthy. Here we describe nine additional species with clear morphological variation coupled with shared characters suggesting a significant radiation of closely related stem-group ants – the first such instance known from Cretaceous ant fauna.

Burmese amber, also called “burmite”, once thought to be Miocene in age [12], is now known to be 99 myo based on radiometric dating [13]. This amber has been commercially exploited for millennia, the earliest record corresponding to ornate burmite carvings from as early as 600 BC [14]. Over the past decade there has been a revived, acute interest in the insect inclusions in burmite, both scientific and personal, largely because it preserves the most diverse Cretaceous paleofauna in amber relative to other Cretaceous amber deposits [9] [15]. The amber is hard and polishes well, it is abundant, and so it can be commercially marketed. In many cases the scientific endeavor to describe and interpret this paleofauna aligns with the interests of private collectors, allowing for the study of otherwise inaccessible material (e.g., Barden and Grimaldi, 2013). Indeed, natural history museums are replete with specimens that were originally in private collections, such as the Frick collection of fossil mammals, the Rothschild collection of birds, and the Henry Edwards collection of Lepidoptera at the American Museum of Natural History. The specimens reported here represent the largest assemblage of Cretaceous ant specimens known to date.

## Materials and Methods

All specimens were originally mined from the Hukawng Valley located in Kachin state, Republic of the Union of Myanmar [16]. Radiometric ^206^Pb/^238^U dating has demonstrated that the strata bearing this amber is Cenomanian in age (98.79±0.62 Ma) based on zircon crystals found within the matrix [17]. Specimens described here were sold to dealers in the region and ultimately purchased by Mr. James Zigras, who kindly provided them for study and allowed preparation of the specimens by trimming and polishing. The specimens are on long term, indefinite loan from James Zigras and available for study to researchers through the American Museum of Natural History, they are: JZC Bu105A, JZC Bu106, JZC Bu108A, JZC Bu111, JZC Bu112, JZC Bu114, JZC Bu115A, JZC Bu121A, JZC Bu222A, JZC Bu223B, JZC Bu223A, JZC Bu224, JZC Bu225, JZC Bu300A, JZC Bu301, JZC Bu302A, JZC Bu303A, JZC Bu303B, JZC Bu304, JZC Bu305, JZC Bu324A, JZC Bu324B, JZC Bu343, and JZC Bu1648.

Burmese amber is relatively hard and tough, it does not readily fracture or splinter compared to many other Cretaceous ambers. Amber pieces were marked on the surface for trimming, so as to maximize dorsal, lateral, and frontal views of each ant inclusion. This was not always possible, particularly when appendages or other inclusions prevented such trimming. Otherwise, the amber was often trimmed 1–3 mm from the surfaces of the ant inclusion. Trimming used a small, water-fed trim saw with a 1 mm-thick diamond-encrusted blade 10 cm in diameter. Flat, trimmed surfaces were then ground slightly and polished using circular, wet emory papers of decreasing grit sizes (400, 600, 800, 1200, 2400, 4000) on a lapidary wheel (Buehler). Embedding in a high quality synthetic resin was necessary only for a few pieces with fractures, following the procedure of Nascimbene and Silverstein [17] (in this study EpoTek 301–2 was instead used as the embedding resin). In order to optimize the observation and measurement of particular structures and views of the ant, the prepared amber piece was lightly pressed into a small (4–5 mm diameter) ball of dental wax or plasticene in the desired orientation. A small drop of glycerine was applied to the upper surface of the amber piece and covered with a glass coverslip, which obscures fine surface imperfections and improves resolution at higher magnifications. Study was generally made using a Leitz Wetzlar stereoscope at magnifications between 48– 144×. Measurements and photomicrographs were made using a Nikon SMZ1500 stereoscope with Nikon NIS software; photomicrographs were z-stacked.

All measurements were recorded in millimeters. Length and width measurements were taken at their greatest value unless otherwise noted. Due to variable preservation, measurements were taken for the holotype only with exception of total body length, which was obtained for paratypes if possible. Paratype and holotype body lengths were reported as a range in descriptions. Mesosomal length was characterized in two ways: a traditional measurement known as Weber’s length taken as a straight line from the anterior margin of the pronotum (excluding any “neck”) to the posteroventral margin of the propodeum; and as individual measurements of the pronotum (including any neck-like anterior extension abutting the occipital carina), mesonotum, metanotum, and propodeum in lateral view. The pronotal, mesonotal, and metanotal measurements were taken as a straight line along the dorsal margin of each sclerite while the propodeum was measured from the anterodorsal margin of the sclerite to the dorsal-most point of anterior petiole attachment. The additional mesosomal measurements were recorded to better capture the relative proportions of individual sclerites and because some specimens were posed in a manner that artificially decreased Weber’s length due to positioning or angle of view (such specimens are noted in descriptions). In cases where exact measurements were not possible due to refractive distortion the structures were not measured, or they were described with approximate relative sizes.

### Nomenclatural Acts

The electronic edition of this article conforms to the requirements of the amended International Code of Zoological Nomenclature, and hence the new names contained herein are available under that Code from the electronic edition of this article. This published work and the nomenclatural acts it contains have been registered in ZooBank, the online registration system for the ICZN. The ZooBank LSIDs (Life Science Identifiers) can be resolved and the associated information viewed through any standard web browser by appending the LSID to the prefix “http://zoobank.org/”. The LSID for this publication is: urn:lsid:zoobank.org:pub:7B4FB94C-DB6F-4441-9414-70CCA9F7431B. The electronic edition of this work was published in a journal with an ISSN, and has been archived and is available from the following digital repositories: PubMed Central, LOCKSS.

## Results

### Genus *Sphecomyrmodes* Engel and Grimaldi

*Sphecomyrmodes* Engel and Grimaldi, 2005:p. 5. Type species: *S. orientalis* Engel and Grimaldi, by original designation and monotypy.

Worker Diagnosis (revised): Species possess a well developed metapleural gland opening and sting; distinct petiole; and shortened scape (<0.25 the total antennal length) like other Sphecomyrminae; differentiated most easily by the small, stout setae along the entire anterior margin of the clypeus. In addition, all known *Sphecomyrmodes* possess bidentate mandibles, and a subapical tooth on the pretarsal claw. Similar clypeal structures are found in one other sphecomyrmine genus and two Cretaceous genera *incertae sedis*. In the case of *Zigrasimecia*, the clypeal setae are accompanied by long, tapered setae on the labrum, which are absent in *Sphecomyrmodes*. The *incertae sedis* genera, *Myanmyrma* and *Gerontoformica*, can be distinguished from *Sphecomyrmodes* by a conspicuous medial gap between the clypeal setae and an elongated scape, respectively.

Composition: Species *contegus* n. sp., *gracilis* n. sp., *magnus* n. sp., *orientalis*, *pilosus* n. sp., *rubustus* n. sp., *rugosus* n. sp., *spiralis* n. sp., *subcuspis* n. sp., *tendir* n. sp., in amber from northern Myanmar, Albian-Cenomanian (ca. 100 myo); species *occidentalis* in amber from Charente-Maritime, France (Early Cenomanian, ca. 100 myo).

***Sphecomyrmodes contegus,*** **new species**

urn:lsid:zoobank.org:act:546C9D7A-AB77-4ACE-851D-203413755CB1 

Figures 1A–E.

**Figure 1:**
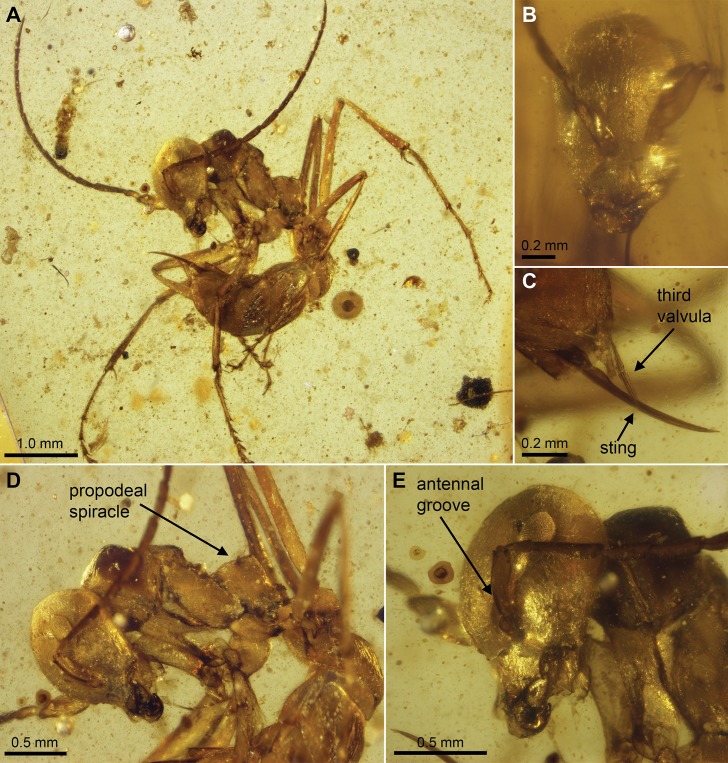
*Sphecomyrmodes contegus* holotype JZC Bu300A photomicrographs. A. Lateral view of entire specimen. B. Face-on view of head. C. Sting with third valvula visible. D. Lateral close-up view of body. E. Lateral close-up view of head and pronotum.

Worker Diagnosis: Distinguished by the presence of antennal scrobes; head elongate, ∼0.50× longer than wide; ocelli highly reduced; metanotal and propodeal spiracles protruding, appear pointed. Gaster segments I and II (abdominal segments III and IV) with slight dorsal constriction between them. Body length 5.05–5.19 mm.

Description: **Head**: Very finely rugose; elongate anteroposteriorly, 1.21 mm in length from posterior margin of head to anterior margin of clypeus; 0.79 mm at widest point just below eyes in frontal view. Ocelli highly reduced, virtually lost. Head capsule cuticle slightly rugose with fine anteroposterior grooves. Occipital carina circular, with smooth edges. Dorsal margin of head nearly flat, very slightly rounded. Center of eyes at 0.70× the distance above anterior margin of clypeus to occipital foramen; eye elongate, 0.28 mm length, 0.17 mm greatest depth. Distance between inner margins of eyes 0.65 mm, eyes bulging slightly in frontal view. Cuticle between antennae raised, narrow; antennal scrobe depth approximately the same as scape diameter, running from just above clypeus to lower margin of eye for approximately 0.30 mm. Antenna total length 3.52 mm: scape 0.34, pedicel 0.15, funicular segment I 0.37, II 0.25, III 0.28, IV 0.29, V 0.29, VI 0.30, VII 0.29, VIII 0.28, IX 0.28, X 0.40; scape broad, 0.16 mm wide [although likely a partial result of desiccation]. Clypeus with concave anterior margin, convex posterior margin meeting linear lateral margins at a 100° angle just below antennae with tentorial pits just above. Clypeus 0.23 mm long in frontal view; entire anterior margin of clypeus with ca 22 stout (0.02 mm long) pointed setae. Anterolateral margin of clypeus raised slightly as a shelf above mandible base. Mandibles falcate, approximately 0.40 mm in length, with two denticles at apex; apical tooth longer than subapical one, approximately half as broad as subapical tooth [lengths difficult to measure as preserved]; external surface of mandible with sparse setae. Labial palps with two equal-sized segments, maxillary palps four-segmented, first approximately 0.3× the length of segments 2, 3, and 4 individually.

**Mesosoma**: Cuticle rugose where detail visible. Aside from pronotum, propleuron, and legs, mesosomal segments dessicated and appear flattened in dorsal view. Weber’s length 1.65 mm. (some flexibility in mesosoma apparent, reducing the length of this metric). Dorsal lengths: Pronotum 0.76 mm, mesonotum 0.59, propodeum 0.64. Pronotum with distinct neck, hourglass-shaped when viewed from above, 0.26 mm wide at head attachment, 0.14 mm in “neck” extension, 0.28 mm at widest point above proxocoxae; in lateral view pronotum with blunt dorsal projection. Dorsal and posterior margins of pronotum almost perfectly linear from lateral view, posterodorsal edge of pronotum a right angle. Pronotal-propleural sulci faint; propleuron reduced, only slightly visible. Mesonotum a trapezoid when viewed laterally, raised above the pronotum and metanotum, the anterior face approximately 0.3× the length of the posterior. Metanotum a distinct sclerite possessing protruding spiracle opening; metanotal groove present as small area of darkened cuticle. Propodeal spiracle visible as oval-shaped opening at the apex of pointed, cuticular projection. Metapleuron covered with small, tapered setae; metapleural gland opening visible, appears as a slight indentation just anterior to petiole attachment. Procoxa with line of small setae along anterior edge, 0.49 mm long, 0.22 mm wide at base, 0.15 mm at lower joint; protrochanter 0.18 mm long, 0.13 mm at widest; profemur 0.89 mm long, 0.15 mm wide, covered in small setae becoming more dense apicad, trochantellus present; protibia 0.93 mm long, 0.10 mm wide, dense patch of setae at lower joint; protibial spur with small subapical projection forming forked tip. Mesocoxa 0.43 mm long, width distorted; mesotrochanter 0.21 mm in length, 0.12 mm wide; mesofemur 1.23 mm long, 0.16 mm wide, small patch of setae at lower joint, trochantellus visible; mesotibia 1.10 mm long, 0.10 mm wide, four stiff, pointed setae and patch of short, tapered setae present at lower joint; two mesotibial spurs present, one slightly pectinate. Metacoxa 0.39 mm in length, width distorted; metatrochanter 0.17 mm in length, width distorted. Meta-femur 1.54 mm in length, 0.14 mm wide, setose near lower joint, trochantellus present; metatibia setose throughout, 1.37 mm in length, 0.10 mm in width; two metatibial spurs present, both finely pectinate. Tarsal segments densely setose, each terminating with four thick, pointed setae. Tarsal claw possessing small subapical point in addition to terminal tooth, resulting in two-pronged claws.

**Mesosoma**: Petiolar-propodeal attachment point obscured, nodiform, pedunculate; petiole approximately 0.50 mm total length (peduncle 0.15 mm). Node evenly rounded with setae along dorsal margin. Helcium distinct segment with clear sulci, 0.13 mm long, attaching to gastral segment I (abdominal segment III) at 0.12 mm in height from lateral view. Abdominal segments 3–7 appear fully extended and possess anteroposterior cuticular grooves along sternites. Slight dorsal constriction between gastral segments I and II (abdominal segments III and IV). Gastral segment I 0.29 mm in length; possessing pointed ventral projection 0.05 mm long. Gastral segment II 0.34 mm in length, gastral segment III 0.28 mm, gastral segment IV 0.18 mm, gastral segment V 0.25 mm, with setose patch on posterior-most region. Hypopygium setose, sting extended, 0.80 mm exposed. Third valvula faintly visible and setose.

Types: Holotype JZC Bu300A. Wingless female (presumed worker). Preserved in a 9×12×3 mm section of transparent yellow amber with a small midge. Some measurements were not possible due to shrinkage and desiccation during preservation. Paratype JZC Bu115-A.

Etymology: From the Latin contego meaning to conceal or protect. Referencing the antennal scrobes, which presumably functioned as reservoirs for antenna scapes as in modern ants.

Comments: Paratype JZC Bu115A may be a different species since it has reduced antennal scrobes and the head is less elongate; preservation, however, makes this difference uncertain.

***Sphecomyrmodes gracilis,*** **new species**

urn:lsid:zoobank.org:act:17A050DD-E97B-49E0-963A-5A48F895C3D3 

Figures 2A–C.

**Figure 2:**
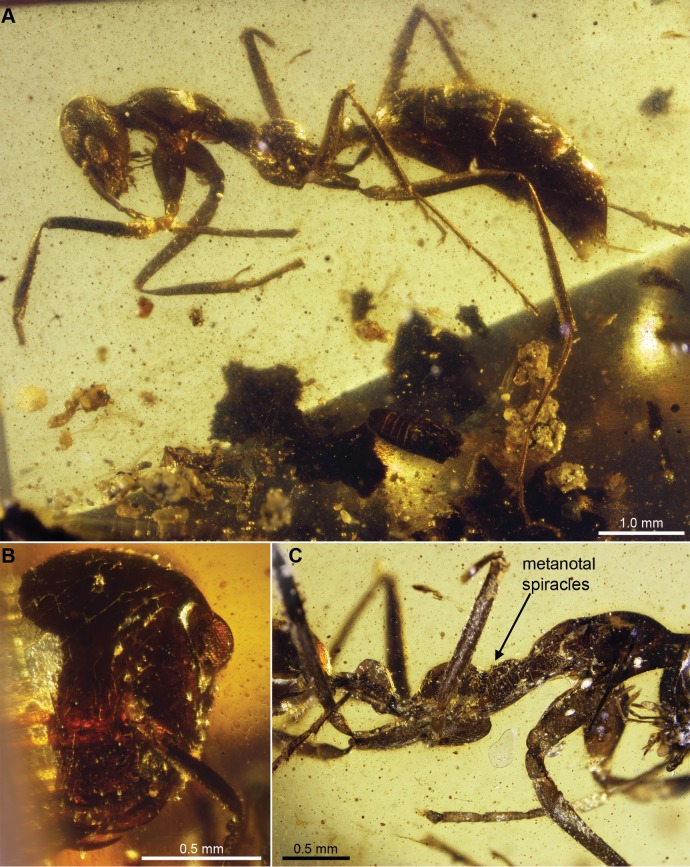
*Sphecomyrmodes gracilis* holotype JZC Bu324A photomicrographs. A. Lateral view of whole specimen. B. Frontal view of partially damaged head. C. Lateral view of elongate mesosoma, petiole, and first sclerite of gaster.

Worker Diagnosis: Dorsal margin of clypeus with short, tapered setae; frontal carina spiraling from antenna base; mesosoma extremely elongate (total length approximately 7 times greater than height at mesonotum/mesopleuron); ocelli small (0.02 mm wide); petiole with small, rounded ventral projection toward anterior margin; gaster long (>3.2 mm). Body length 6.62 mm.

Description: **Head**: [One side of head partially missing, lost at surface of amber]. Head with sparse, tapered setae. Head capsule gradually rounded at posterior and anterior ends in frontal view, length 1.14 mm from posterior margin of head to anterior margin of clypeus, greatest width 1.10 mm. Head in profile drop-shaped, 0.54 mm deep at occipital carina; 0.70 mm depth from median axis of eyes, 0.30 mm depth from anterior margin of clypeus. Ocelli present, situated just above posterior margin of eyes, circular, approximately 0.02 mm diameter, slightly obscured by fractures in cuticle. Midline of eye positioned 65% of the distance from anterior margin of clypeus to occipital foramen, bulging in anterior view, inner margins approximately 0.80 mm apart; eye 0.33 mm deep, 0.20 mm wide. Cuticle raised between antennal bases; bases embedded but exposed, internal margin with small, curved, frontal carina running to just lateral to anterior margin of eyes; tentorial pits present just below anterior terminus of frontal carina. Scape 0.48 mm, pedicel 0.18, funicular segment I 0.42, II 0.34 mm, III 0.31, IV 0.35, V 0.35, remaining segments missing; scape broad at base (0.15 mm), abruptly narrowed to 0.07 mm. Clypeus 0.22 mm long, posterior margin concave, anterior margin convex, lateral margin linear and meeting posterior suture at 145° angle. Anterolateral margin of clypeus covers mandible base, slightly upturned; dorsal surface covered with short, tapered setae. Entire anterior margin of clypeus with row of numerous (>20) small, pointed, peg-like setae. Mandible with two denticles, apical tooth slightly larger of the two. [Mandibular measurements not possible as preserved]. Maxillary palps with four palpomeres, I 0.14 mm, II 0.20 mm, III 0.20 mm, IV 0.16 mm. Labial palps not visible.

**Mesosoma**: Long and gracile, Weber’s length 2.34 mm; height 0.32 mm, measured as a perpendicular straight line at mesonotum/mesopleuron. Segment lengths in lateral view: pronotum 0.89 mm, mesonotum 0.57 mm, metanotum 0.28 mm, propodeum 0.69 mm. Anteriorly, pronotum extends into neck, in dorsal view appears as a rounded rectangle meeting occipital carina; carina oval in dorsal view; pronotum gradually widens posteriad from 0.28 mm at head attachment point to 0.55 mm above procoxae. Pronotal-propleural sulci well developed. Mesonotum and mesopleuron very narrow in lateral view, greatest depth 0.12 mm and 0.21 mm, respectively. Mesopleuron extremely long, 0.77 mm, widely separating fore- and mid-legs. Small depression between mesonotum and metanotum, which are at same dorsally height, both slightly rugose. Metanotal spiracle opening small and turret-like, surrounding cuticle dorsally pointed. Metanotal groove a distinct impression. Propodeum bulbous, dorsal surface gradually rounded, propodeal spiracle raised onto apices of two small, blunt cuticular projections near metanotal groove. Metapleural gland opening visible above metacoxa as a slight oval-shaped indentation. Legs with sparse, tapered setae. Cuticle on legs darkened, trochantellus not observable. Procoxa 0.71 mm long, 0.38 mm wide at base, 0.18 mm at apex; protrochanter 0.20 mm long, 0.15 mm wide; profemur 1.28 mm long, 0.20 mm wide; protibia 1.13 mm long, 0.13 mm wide, with single spur having subapical point [protarsi lost from specimen]. Mesocoxa 0.42 mm long, width unclear; mesotrochanter 0.18 mm long, 0.14 mm wide; mesofemur 1.12 mm long, 0.17 mm wide; mesotibia 1.26 mm long, 0.12 mm wide, with two apical spurs, of equal length. Metacoxa 0.52 mm long, width obscured; metatrochanter 0.26 mm long, 0.14 mm wide; metafemur 1.59 mm long, 0.16 mm wide; metatibia 1.71 mm long, 0.17 mm wide; pair of metatibial spurs, each simple and of equal length. Tarsi terminate with six pointed setae at apex. Meso- and metatarsal claws with subapical tooth.

**Metasoma**: Petiole nodiform, with peduncle short (0.15 mm), total length 1.00 mm, attached to propodeum at a height of 0.15 mm in lateral view, broadened to 0.52 mm at apex of node; node rounded. Immediately ventral to anterior margin of petiole node is ventral projection 0.12 mm high, gradually rounded at apex. Helcium with distinct anterior and posterior sulci, length 0.20 mm, attaches to gaster segment I (abdominal segment III) at a height of 0.18 mm. Gaster elongate, segment I length 0.60 mm, II 0.84 mm, III 0.80 mm, IV 0.52 mm, V 0.47 mm. Sternite of segment I with pointed ventral projection 0.08 mm long. Pygidium and hypopygidium with tapered setae projecting posteriad. Sting extruded, 0.53 mm exposed. Third valvula faintly visible, setose.

Types: Holotype JZC Bu324A. Wingless female (presumed worker). Amber a clear yellow, trimmed to 12×9×4 mm. Also preserved are particles of detritus and a disembodied beetle head. Paratype JZC Bu324B.

Etymology: From Latin “gracilis” meaning slender in reference to the elongated nature of the species.

***Sphecomyrmodes magnus,*** **new species**

urn:lsid:zoobank.org:act:36FBF68D-B35C-48E2-8B15-81505E22D08F 

Figures 3A–D.

**Figure 3:**
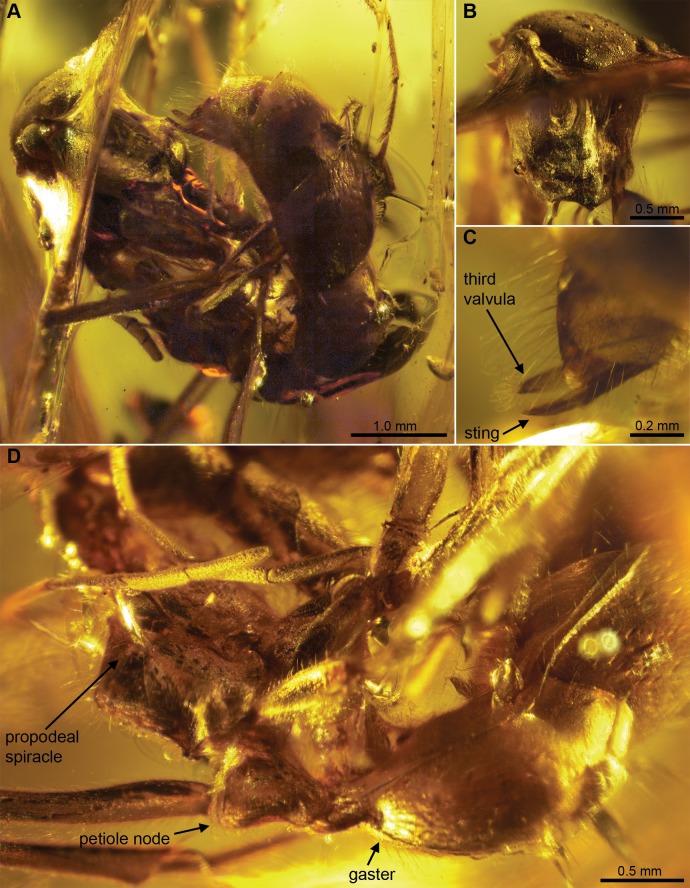
*Sphecomyrmodes magnus* holotype JZC Bu108A photomicrographs. A. Lateral view of entire large specimen. B. Face-on view of head. C. Lateral view of sting and third valvula. D. Dorsolateral view of gaster, petiole, and propodeum.

Worker Diagnosis: Body size very large, approximately 1.7× size of other species, on average; body length 8.03–8.64 mm; head capsule slightly elongate from frontal view 1.25× longer than wide; ocelli clearly visible; petiole sessile; darkened and prominent metanotal groove.

Description: **Head**: Elongate in frontal view, length 1.49 mm from anterior margin of clypeus to posterior margin of head, width 1.18 mm below eyes. In profile, head capsule drop-shaped, narrowing toward anterior end, with cuticular buldge at medial axis. Head somewhat flattened dorsally from medial margin of eyes to occipital carina, gradually rounded at apex. Occipital carina oval, gradually rounded with no apparent deviations in shape. Eyes very large, 0.44 mm long, 0.35 mm wide; high on head capsule, at 80% of the distance from anterior margin of clypeus to occipital foramen; distance between inner margins of eyes wide, 0.95 mm; eyes bulging significantly in frontal and dorsal views. Ocelli present, oval, 0.10 mm long, 0.06 mm wide, spaced 0.17 mm apart on flattened cuticle between posterior margin of eyes. Cuticle raised between antennae. Antennal bases exposed, 0.15 mm in diameter, seated in oval cuticular depressions approximately 0.50 mm long, 0.40 mm wide; tentorial pits present near anterior margin of these depressions; depressions surrounded along interior curvature by frontal carina, which runs from 0.11 mm below antenna base to ∼0.20 mm below anterior margin of eyes. Antenna: total length 6.66 mm, scape 0.70, pedicel 0.18, funicular segment I 0.61, II 0.51, III 0.65, IV 0.64, V 0.62, VI 0.58, VII 0.57, VIII 0.50, IX 0.48, ×0.62. Funicular segments setose and punctate. Clypeal sclerite with posterior margin concave and anterior margin convex, lateral margins linear, meeting posterior sulci at ∼130° angle. Clypeus 0.44 mm long at middle, possessing slightly upturned anterolateral shelf above mandible base; with long, tapered setae (up to 0.24 mm) on dorsal surface and anterior margin. Entire anterior margin of clypeus protruding, rounded, with comb of very stout (∼0.02 mm long), pointed setae (difficult to enumerate as preserved but >25). Mandibles obscured by fissure, appear narrowed for apical 2/3, possibly bidentate. Maxillary and labial palps not visible.

**Mesosoma**: Robust, of similar height and length; setose, especially on dorsal surface. Weber’s length 2.51 mm (angle of view likely reducing this measurement slightly). Segment lengths in lateral view: pronotum 1.20 mm, mesonotum 0.63, metanotum 0.29, propodeum 0.90 mm. Pronotum with anterior extension, terminus rounded to meet and accommodate rounded occipital carina; extension concealed in lateral view, sclerite gradually rounded dorsally. Pronotal-propleural suture well developed, propleuron only slightly exposed. Pronotal cuticle concave laterally just above procoxa. Mesopleuron broad, 0.88 mm greatest width. Dorsal surface of metanotum lower than mesonotum and propodeum; metanotal groove visible as darkened region. Propodeum shaped as a rounded right angle posterodorsally; spiracle opening near anterior sulci, protruding with surrounded cuticle pointed; patch of long, tapered setae (up to 0.17 mm) on dorsal surface; metapleural gland opening visible as small pit just above metacoxa. Legs thick, long, covered in tapered setae of varied lengths. Procoxa 0.95 mm long, 0.50 mm wide at base, 0.36 mm wide at apex; protrochanter 0.38 mm long, 0.18 mm wide; profemur 2.19 mm long, 0.26 mm wide, trochantellus visible; protibia 1.65 mm long, 0.22 mm wide; Protibial spur not pectinate, flanked by two small stiff setae approximately 0.20 and 0.10× spur length. Mesocoxa 0.77 mm long, 0.43 mm wide at base, 0.20 mm at apex; mesotrochanter 0.42 mm long, 0.09 mm wide at base, 0.22 mm at apex; mesofemur 2.41 mm long, 0.27 mm greatest width, trochantellus present; mesotibia 2.34 mm long, 0.24 mm wide; two mesotibial spurs of equal length, both appear simple, ring of 12 stiff setae approximately 0.20× spur length at apex of mesotibia. Metacoxa 0.68 mm long, width distorted; metatrochanter 0.39 mm long, 0.14 mm wide at base, 0.19 mm at apex; metafemur approximately 2.24 mm long, 0.20 wide, trochantellus visible; metatibia 2.03 mm long, 0.23 mm wide; two metatibial spurs of equal length present, one simple, one pectinate. Tarsus setose throughout, each tarsomere terminating with 6 prominent, pointed setae, pretarsal claw with subapical tooth.

**Metasoma**: Petiole approximately 0.85 mm long, sessile, attaches broadly (0.37 mm in lateral view) to propodeum; dorsal surface with sparse setae. Node rounded dorsally with flattened lateral surfaces; petiole height gradually increased to 0.62 mm at apex, spiracle clearly visible in center. Helcium with distinct sulci, 0.26 mm in length, attaching to gaster segment I (abdominal segment III) at height of 0.27 mm; segment I 1.14 mm long, segment II 0.56, III 1.30, IV 0.61, V 0.36, however, elongated segment I a result of disarticulation. Gaster slightly setose; segments III, IV, V with additional, numerous, tapered setae at posterior end. Pygidium setose; with blunt, setose third valvula visible 0.18 mm long, 0.08 mm wide, just above sting. Sting extruded 0.25 mm.

Types: Holotype JZC Bu108A. Wingless female (presumed worker). Preserved in 15×9×6 mm section of clear yellow amber – no other major inclusions. Paratypes JZC Bu114, JZC Bu343.

Etymology: From the Latin “magnus” meaning large, in reference to the striking size of this species.

***Sphecomyrmodes pilosus*, new species**

urn:lsid:zoobank.org:act:4EB90F51-E862-4AEC-A7D7-8F104AF459A2 

Figures 4A–C.

**Figure 4:**
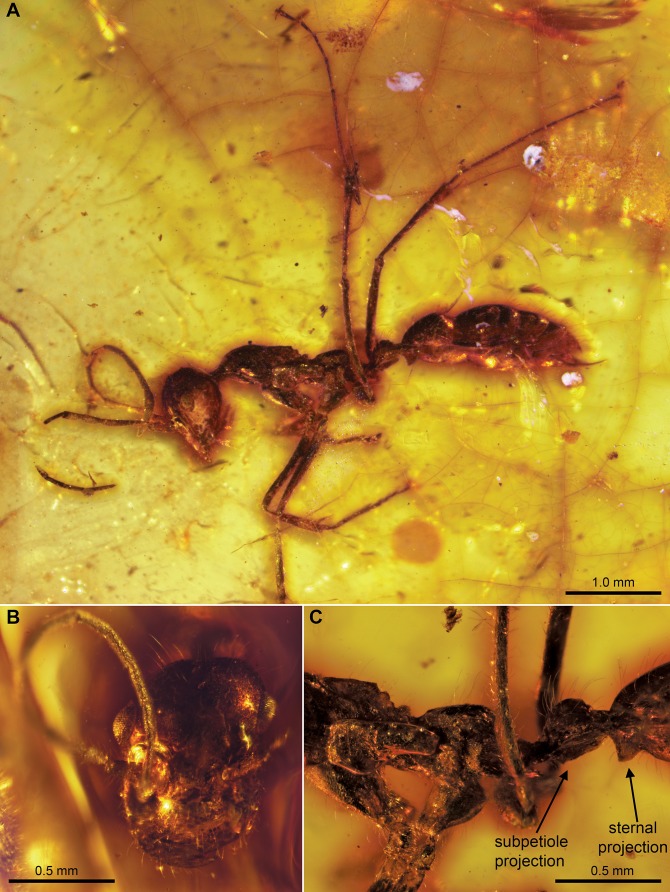
*Sphecomyrmodes pilosus* holotype JZC Bu225 photomicrographs. A. Lateral view of entire specimen. B. Frontal view of head. C. Lateral view of posterior region of mesosoma and anterior portion of metasoma.

Worker Diagnosis: Distinguishing features include high developed pilosity over much of body, with setae as long as ∼0.25 mm; pronotum elongated into narrow neck; metanotal and propodeal spiracles protruding approximately 0.04 mm; petiole with flattened, long, ventral projection; sternal projection on gaster segment I (abdominal segment III) greater than 0.10 mm and slightly hooked; gaster segment I and II with narrow but deep dorsal constriction between them. Approximate body length 4.31 mm.

Description: **Head**: Slightly elongate, length 0.91 mm from anterior margin of clypeus to posterior margin of head, 0.69 mm width in frontal view. Head capsule teardrop-shaped in profile, 0.58 mm at widest point just posterior to eyes, narrowing to 0.23 mm at anterior margin of clypeus. Ocelli minute, diameter 0.03 mm, but significantly raised above surface of surrounding cuticle. Occipital foramen completely flat along medial axis. Dorsal surface of head with long, tapered setae in patch between ocelli and occipital foramen. Occipital carina not clearly visible, appears to be oval with smooth edges. Eye 0.18 mm in length, 0.14 mm wide, inner margins far apart (0.64 mm); eyes bulging laterally when viewed frontally, set approximately 2/3 from anterior edge of clypeus to occipital foramen. Stout, tapered setae present on gena along posteroventral face of head. Cuticle raised between antennal sockets, antennal bases exposed, surrounded posteriorly by semi-circular frontal carina reaching just below compound eyes. Total antenna length 2.61 mm.; scape 0.35, pedicel 0.15, funicular segment I 0.33, II 0.19, III 0.20, IV 0.19, V 0.20, VI 0.17, VII 0.17, VIII 0.20, IX 0.17, ×0.29; scape highly setose, remaining segments with sparse setae. Anterior and posterior clypeal margins convex and concave respectively; posterior margin meeting linear lateral margin at a 120° angle; tentorial pits present just above lateral and posterior clypeal margin union. Clypeal sclerite covered in tapered setae of various lengths ranging from 0.04 to 0.24 mm. Entire anterior margin of clypeus with row of ca 20 short, stout setae approximately 0.02 mm long. Mandible bidentate, falcate, 0.41 mm long from base to apical tooth; apical tooth 0.29 mm long; basal tooth 0.15 mm, nearly twice the width of apical tooth at base; inner and outer surfaces of mandible with fine, sparse setae. Labial palps with two equal-sized palpomeres; maxillary palps with four segments, the fourth nearly twice the length of first three individually; all palpomeres densely covered with short setae.

**Mesosoma:** Setae present throughout, especially lengthy along dorsal margins. Mesonotum elongate, narrow overall; Weber’s length 1.56 mm. Pronotum extended anteriad into a narrow neck; total length 0.70 mm as measured dorsally (neck comprising 0.29 mm). Dorsal length of mesonotum 0.55 mm; metanotum 0.21 mm, propodeum 0.55 mm. Propleural sulci well developed, ventral surface of propleuron covered with short, tapered setae; mesonotum with sparse setae of varied lengths. Mesonotum finely rugose, setose, surface gradually sloped. Metanotum with blunt, turret-like spiracle opening; sclerite itself covered in coarse, erect setae. Propodeum dorsally rounded with setae 0.15 mm long. Propodeal spiracle situated at top of small, pointed, cuticular projection. Metapleuron faintly rugose; metapleural gland opening visible as small hole above metacoxae. Legs long, with each segment covered in setae. Procoxa 0.43 mm long, 0.33 mm wide at base, 0.14 mm at lower joint; protrochanter 0.14 mm long, 0.14 mm widest point; profemur 0.92 mm long, 0.10 mm trochantellus not visible; protibia 0.67 mm long, 0.10 mm wide, tibial spur with small subapical tooth. Mesocoxa 0.27 mm long, 0.21 mm wide at base, 0.14 mm at lower joint; mesotrochanter 0.16 mm long, 0.11 mm wide; mesofemur 0.87 mm long, 0.10 mm wide, trochantellus visible; mesotibia 0.86 mm long, 0.09 mm wide, with two tibial spurs of equal length. Metacoxa 0.31 mm in length; metatrochanter 0.12 mm long, 0.08 mm wide; metafemur 1.11 mm long, 0.06 mm wide, trochantellus visible; metatibia 0.78 mm long, 0.09 mm wide, Tibial spur pectinate, accompanied by stiff, pointed setae on tibia approximately 0.3× length of spur. Tarsus heavily setose, apex of each tarsomere with whorl of four distinct, stout setae. Apex of distitarsomere with stout setae radiating in multiple directions. Pretarsal claw with subapical tooth.

**Metasoma**: Petiole nodiform and extended, length 0.55 mm (peduncle comprising 0.11 mm), attaching to propodeum at height of 0.12 mm from lateral view. Peduncle with single small (<0.02 mm), narrow, pointed, dorsal projection. Dorsal node rounded and prominent, 0.16 mm high, possessing long setae up to 0.15 mm. Flat, ventral projection runs length of dorsal node, projecting ca 0.3× the height of node in opposite plane; spiracle visible in anterior region of this projection. Helcium not visible. Petiole attaches to gastral segment I (abdominal segment III) at height of 0.14 mm in lateral view. Gaster elongate, first two segments fully extended; long, sparse setae present on tergites and sternites throughout. Gastral segment I (abdominal segment III) 0.52 mm in length, heavily constricted dorsally where meeting segment II; with large (0.11 mm), pointed, slightly hooked sternal projection. Gastral segment II length 0.63 mm, segment III 0.28 mm, segment IV 0.17 mm, V 0.21 mm. Sting extruded, 0.60 mm exposed.

Types: Holotype JZC Bu225. Wingless female (presumed worker). Preserved in dark-yellow amber trimmed to 12×12×3 mm, accompanied by particles of detritus; amber is also cracked and darkened near inclusion.

Etymology: From the Latin pilosus meaning hairy. Referring to the presence of setae over much of the body.

***Sphecomyrmodes robustus,*** **new species**

urn:lsid:zoobank.org:act:953302E0-AC6D-44FB-BC15-734C3A29D371 

Figures 5A–C.

**Figure 5:**
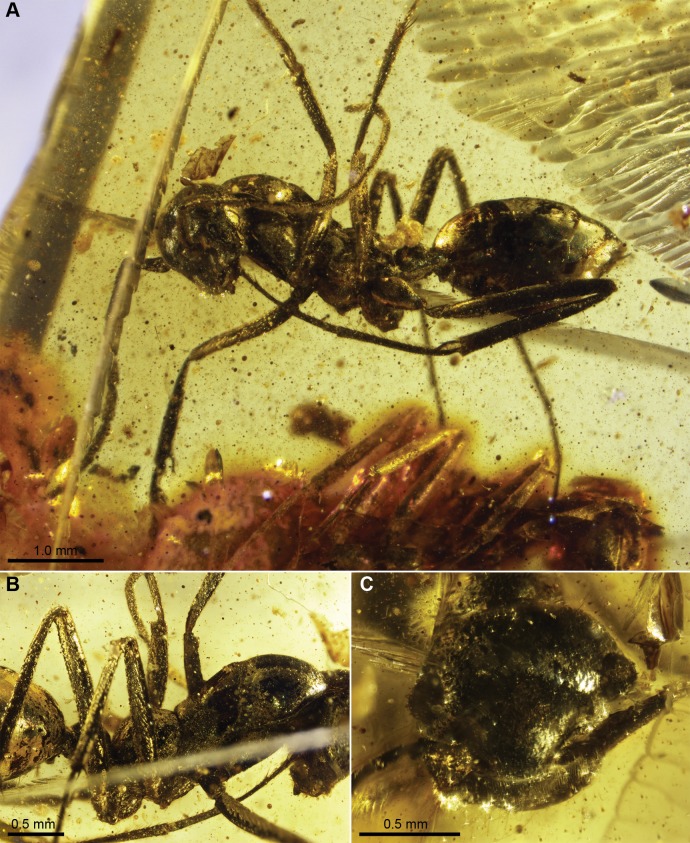
*Sphecomyrmodes robustus* holotype JZC Bu223A photomicrographs. A. Full lateral view of specimen. B. Lateral view of mesosoma, petiole, and first gastral sclerite. C. Frontal view of head.

Worker Diagnosis: Head dorsally flattened, frontal lobe present; mesosoma robust (height approximately 30% of length); metanotum with surface smooth; procoxae very broad (0.49 mm), protibial spur very thick, metapleural gland opening cavernous; petiole sessile. Body length 4.07–5.70 mm.

Description: **Head**: Square, gradually rounded at each margin, length from anterior margin of clypeus to posterior margin of head 0.98 mm, width below eyes 0.95 mm. Area between eyes flattened, extended anteriorly between antennae in form of a broad, rounded frontal lobe. Occipital carina a rounded rectangle, broad, 0.36 mm. Ocelli small, 0.04 mm in diameter, equidistant to each other, located between posterior margins of eyes on flattened surface of head. Distance between inner margins of eyes 0.78 mm; midline of eye located at 65% of distance between anterior margin of clypeus and occipital foramen; eye nearly round, 0.28 mm deep, 0.24 mm wide. Antennal socket oval, 0.12 mm×0.07 mm, embedded in raised frontal lobe but visible in frontal view; tentorial pits visible just anterior to antenna base. Frontal carina extends from anterior margin of frontal lobes to below anterior margins of eyes, curving outward. Torulus raised to obscure approximately half of antennal base; antennal length 4.22 mm; scape 0.46 mm, pedicel 0.15 mm, funicular segment I 0.46 mm, II 0.32, III 0.35 mm, IV 0.37, V 0.37, VI 0.31, VII 0.35, VIII 0.33 IX 0.34, ×0.41, antennomeres devoid of long setae. Gena covered with fine, tapered setae. Frontal lobe terminates at posterior margin of clypeus. Clypeus length 0.23 mm along midline, surface raised slightly, anterior margin of clyepus convex, posterior margin concave, meeting linear lateral margin at 135° angle. Lateral margin of clypeus upturned slightly, pointed anterior projection conceals mandible base. Clypeal sclerite covered with fine, tapered setae; also with 8–10 long, fine setae (0.20 mm) projecting anteriad. Entire anterior margin of clypeus with row of at least 20 stout setal pegs. Mandible falcate, bidentate, setose [partially obscured, preventing measurements; maxillary and labial palps not visible].

**Mesosoma**: Robust, Weber’s length 2.04 mm; height 0.60 mm, measured as a perpendicular straight line at mesonotum/mesopleuron. Segment lengths, lateral views: Pronotum 0.88 mm, mesonotum 0.58 mm, metanotum 0.21 mm, propodeum 0.62 mm. Pronotum-propleuron sulci well developed; propleuron reduced, hardly visible in lateral view, covered with fine setae. Mesonotum and metanotum with no apparent sculpturing except small projecting metanotal spiracle, glabrous. Metanotal groove narrow, deep, prominent. Propodeum gradually rounded with flattened dorsal face, propodeal spiracle at tip of small, blunt dorsal projection. Metapleural gland opening present, 0.09 mm wide, oval-shaped, above metacoxa. No trochantellus present on any leg pairs. Procoxa 0.67 mm long, 0.49 mm wide at base, 0.22 mm at apex; protrochanter 0.21 mm long, 0.15 mm wide; profemur 1.02 mm long, 0.24 mm wide; protibia 0.92 mm long, 0.14 mm wide, with single, simple spur possessing subapical tooth; protibial spur very thick, 0.05 mm wide at base, accompanied by two stiff pointed setae approximately 0.5× its length. Mesocoxa 0.37 mm long, width obscured; mesotrochanter 0.17 mm long, 0.15 mm wide; mesofemur 1.30 mm, 0.22 mm wide; mesotibia 1.21 mm long, 0.15 mm wide, with one simple and one pectinate spur of equal lengths. Metacoxa 0.67 mm long, 0.31 mm greatest width; metatrochanter 0.26 mm long, 0.14 mm wide; metafemur 1.73 mm long, 0.22 mm wide; metatibia 1.62 mm long, 0.20 mm wide, with two apical spurs, one pectinate, other simple. Tarsi setose, each one terminating with four prominent, pointed setae. Pretarsal claw with subapical tooth.

**Metasoma**: Petiole nodiform, with no apparent peduncle, attaches to propodeum at a height of 0.13 mm. Node gradually rounded dorsally, posteriorly decreasing in height, petiole cylindrical between node and gaster attachment, greatest height 0.40 mm, possessing linear groove along lateral face, attaching to gastral segment I (abdominal segment III) at a height of 0.20 mm. Gastral segment I with small (0.05 mm), slightly hooked projection on sternite. Gaster segments telescoping, retracted. Segment I 0.43 mm long, II 0.85 mm, III 0.42 mm, IV 0.21 mm [segment V retracted and not visible]. Segment IV setose along posterior margin. Sting slightly extruded, 0.18 mm exposed. Third valvula visible, glabrous, appears to be two separate pointed structures.

Types: Holotype JZC Bu223A. Wingless female (presumed worker) in a 18×7×4 mm section of transparent yellow amber. Also contained are particles of detritus, a partial midge and an additional, heavily distorted and decomposed wingless female ant. Paratypes JZC Bu106, JZC Bu223B (other decomposed inclusion in same piece).

Etymology: From Latin “robustus” meaning strong, in reference to thick-bodied nature of the animal.

***Sphecomyrmodes rugosus,*** **new species**

urn:lsid:zoobank.org:act:33FCF64E-65BA-429A-989C-FD0EE86F65E0 

Figures 6A–C.

**Figure 6:**
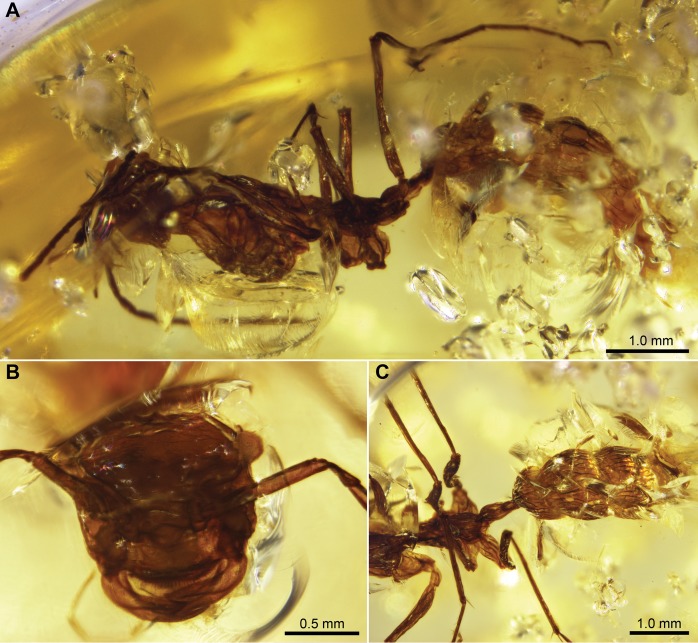
*Sphecomyrmodes rugusus* holotype JZC Bu1648 photomicrographs. A. Lateral view of specimen, which is largely obscured by air inclusions from any single view. B. Frontal view of head. C. Dorsal view of mesosoma, petiole, and gaster.

Worker Diagnosis: Distinguished by distinct, longitudinally rugose sculpturing throughout meso- and metasomal surface. Head ∼1.20× wider than long; cuticle raised between and posterior to antennae; eyes small relative to congeners (depth 0.15 mm, width 0.08 mm). Maxillary palps very prominent, with six palpomeres (as opposed to 4 in other known species). Cylindriform petiole with high propodeal attachment point. Total body length: 4.97 mm.

Description: **Head**: Head rounded-rectangular, wider than long, length from anterior margin of clypeus to posterior margin of head 0.72 mm, width below eyes at greatest 0.90 mm. Occipital carina obscured by bubble inclusion. Ocelli obscured by fissure. Distance between inner margins of eyes 0.64 mm. Midline of eye ∼70% of the distance between anterior margin of clypeus and occipital foramen. Eyes small, elongate, 0.15 mm deep, 0.08 mm wide; bulging when viewed head-on. Cuticle between antennae raised as a fontal lobe, this elevated region begins at posterior clypeal margin and extends posterior to antennae as a notched shelf above antennal bases and clypeus, eyes present at margins of this heightened shelf. Antennal socket oval, 0.08 mm×0.05 mm, surrounded on one lateral margin and posteriorly by raised cuticle. Antennal length 2.64 mm; scape 0.48 mm, pedicel 0.19 mm, funicular segment I 0.27 mm, II 0.22, III 0.22, IV 0.18, V 0.21, VI 0.21, VII 0.20, VIII 0.19, IX 0.19, V 0.26, antennomeres glabrous. Clypeus raised medially, 0.14 mm in length along midline, anterior margin convex, posterior margin concave, suture of lateral margin not visible. Anterolateral margin of clypeus raised and upturned slightly, covers base of mandible. Entire anterior margin of clypeus with over 32 stout (0.02 mm), slightly pointed setal pegs. Just above clypeal pegs are sparse, tapered setae up to 0.06 mm in length. Mandible simple, bidentate, with apical tooth 3× length of basal tooth. Maxillary palp with six palpomeres, basal-most palpomere obscured, second and third palpomeres approximately 60% the length of the remaining three individually. Labial palps somewhat obscured, two equal-sized palpomeres visible.

**Mesosoma**: Pronotal and propodeal cuticle with rugose sculpturing, in the form of deep longitudinal grooves; grooves not visible on other mesosomal segments perhaps due to preservation. Weber’s length 1.63 mm, mesosomal height distorted by desiccated nature of specimen. Segment lengths, from lateral view: Pronotum 0.84 mm, mesonotum-metanotum combined length 0.41 mm, suture not visible due to transverse fissure, propodeum 0.46 mm. Propleuron very narrow from lateral view, propleuron-pronotum sulci well developed. Metanotal spiracle opening produced; metanotal groove visible as region of darkened cuticle. Propodeum possseses large, oval-shaped metapleural gland opening 0.06 mm wide above anterior margin of metacoxa. Propodeal spiracles present at tips of dorsal cuticular projections near anterior suture. Petiole attaches high near dorsal margin of propodeum. No trochantellus present on legs. Procoxa 0.42 mm long, width distorted; protrochanter 0.15 mm long, 0.11 mm wide; profemur 0.85 mm long, 0.14 mm wide; protibia 0.80 mm long, 0.11 mm wide with small, tapered setae near anterior joint and single spur with small subapical point; spur accompanied by a stiff basal seta ∼30% as long as spur itself. Mesocoxa 0.32 mm long, width distorted; mesotrochanter 0.13 mm long, width 0.12 mm; mesofemur 0.63 mm long, 0.13 mm wide with small tapered setae near apex joint; mesotibia 0.97 mm long, 0.09 mm wide, possessing numerous tapered setae near apical joint and single pectinate spur. Metacoxa 0.38 mm long, width distorted; metatrochanter 0.11 mm long, width 0.10 mm; metafemur 0.69 mm long, 0.12 mm wide with small tapered setae at apex; metatibia 1.19 mm long, 0.09 mm wide with numerous tapered setae near apex and paired spurs (one pectinate, one simple) of equal length. Tarsi setose, each with four stiff, pointed setae at terminus; pretarsal claw with subapical tooth.

**Metasoma**: Petiole cylindrical, with slight dorsal projection, longitudinal sculpturing present throughout, attaches to propodeum at a height of 0.09 mm, increasing to height of 0.17 mm at apex of rounded node, total length of 0.47 mm, attaches to gaster segment I (abdominal segment III) at a height of 0.12 mm; no helcium sclerite visible. Gaster segments heavily sculptured and rugose. Gaster tergite segments disarticulated, sclerites not visible as preserved. Gaster segment I 0.29 mm long, II 0.60 mm, III 0.59 mm, segments IV and V largely obscured by fissures.

Types: Holotype JZC Bu1648, wingless female (presumed worker) in transparent yellow piece of amber, rounded and polished to 19×15×4 mm. Specimen surrounded by numerous small bubble inclusions as well as fibrous plant material.

Etymology: From the latin word “rugose” meaning wrinkled, in reference to cuticular sculpturing.

Comments: Specimen is heavily desiccated in some regions, however the characteristic rugosity is unlikely a result of this desiccation as many sclerites, as well as the head capsule, remain more glabrous in appearance. The two additional palpomeres on the maxillary palps is a significant difference, as is the frontal lobe and cylindrical petiole with high attachment.

***Sphecomyrmodes spiralis,*** **new species**

urn:lsid:zoobank.org:act:EBEA426D-72BF-4942-8AE7-58669BB8C5B8 

Figures 7A–C.

**Figure 7:**
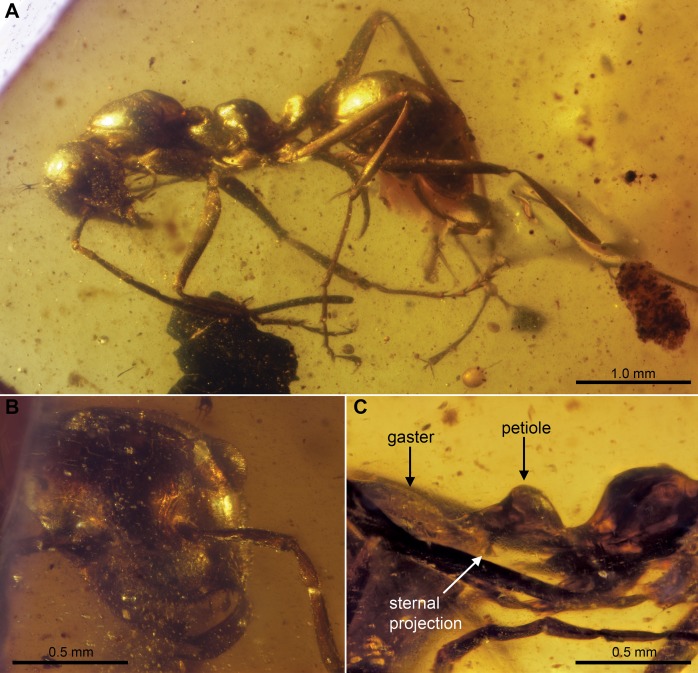
*Sphecomyrmodes spiralis* holotype JZC Bu222A photomicrographs. A. Full lateral view of entire. B. Face-on view of head. C. Lateral view of anterior portion of gaster, petiole, and propodeum.

Worker Diagnosis: Most similar to *S. orientalis* but distinguished by fewer setae on exterior mandible margin surface and presence of sternal projection on anterior margin of gaster segment I (abdominal segment III) – some other characters not observable in *S. orientalis* holotype. Can be separated from other *Sphecomyrmodes* species by sinuous frontal carina spiraling out from antennal base; head slightly drop-shaped in profile view with greatest height/width posteriorly. Body length 4.22–5.11 mm.

Description: **Head**: gradually widened antero-posteriorly and dorsoventrally. Frontal view: width just below eyes ca 1.2× greater than width at clypeus; laterally, depth just below eyes nearly twice depth at clypeus; head width just below eyes 0.90 mm, height from anterior margin of clypeus to posterior margin of head ca 0.90 mm. Head slightly flattened dorsally. Ocelli present, small, 0.05 mm diameter, located between center of eyes when viewed dorsally. Occipital foramen gradually rounded, oval-shaped. Center of eyes positioned above anterior ridge of clypeus at 70% the distance between ridge and occipital foramen; eyes bulging when viewed frontally, inner margins 0.80 mm apart. Eye length ca 1.25× the depth. Antennal bases exposed, oval, longer radius twice that of shorter radius. Cuticle raised between antennae, with semi-circular frontal carina originating at anterior ridges of toruli, curving and terminating lateral to bottom margin of eyes. Total antenna length 3.31 mm: Scape 0.48, pedicel 0.17, funicular segment I 0.35, II 0.26, III 0.25, IV 0.28, V 0.25, VI 0.24, VII 0.26, VIII 0.32, IX 0.18, ×0.27 mm. All segments narrow and devoid of long setae. Clypeus a rounded trapezoid, with convex, parallel anterior/posterior margins and straight lateral margins converging at a 140° angle just below antennae; tentorial pits visible just lateral to this union on head capsule. Clypeus length 0.21 mm along medial axis, sparse tapered setae approximately 0.12 mm in length present on dorsal surface. Clypeal cuticle raised slightly in center and near lateral margins, entire anterior margin possesses ca 25 stout (0.02 mm long) setae. Anterolateral margin of clypeus with slight anterior projection, obscures mandible base when viewed from above. Mandibles falcate, 0.36 mm from base to tip of apical tooth, gradually hooked, with slight groove along inner curvature. Mandibles with two denticles at terminus, apical tooth approximately 1.4× longer and slightly more narrow than basal tooth. Mandibles with sparse setae on outer margin (mandibles preserved slightly extended but appear to retract flush with anterior margin of clypeus). Two labial palpomeres present, basal palpomere slightly longer. Maxillary palps with four segments, basal one 0.3× size of remaining equal-sized segments.

**Mesosoma**: Weber’s length 1.70 mm. Dorsal length of pronotum 0.70 mm, mesonotum 0.41, metanotum 0.20, propodeum 0.52. Dorsally, pronotum broadly attached to ca 0.3× the width of head. From above, pronotum narrows anteriorly to accomodate head capsule concavity, gradually widens 1.5× in width above fore coxae. Pronotum-propleuron sulcus well developed. Mesonotum slightly rugose, sloped at 60° angle to metanotum. Metanotum with distinct sulci surrounding the sclerite, raised slightly above mesonotum and propodeum; metanotal spiracle protruding, surrounding cuticle pointed dorsally. Metanotal groove clearly visible. Propodeum gradually rounded postero-dorsally, propodeal spiracle turret-like. Metapleural gland opening visible as sizeable oval-shaped indentation just above metacoxa. Propodeum with steep (75°) slope leading to petiole. Procoxa 0.73 mm in length, 0.36 mm wide, with many short setae. Protrochanter 0.15 mm length, 0.13 mm wide; profemur 0.94 mm length, 0.16 mm wide with trochantellus visible; protibia 0.75 mm long, 0.11 mm wide. Single protibial spur with small subapical point, spur pectinate between subapical and apical points. Two stiff setae at spur base approximately 0.75× spur length. Mesocoxa 0.27 mm long, 0.15 mm wide; mesotrochanter 0.16 mm long, 0.08 mm wide; mesofemur 1.11 mm long, 0.15 mm wide, with trochantellus and small, tapered setae projecting from lower joint; mesotibia 1.01 mm long, 0.12 mm wide, with one pectinate and one simple tibial spur equal in length, setae approximately half spur length present at lower joint. Metacoxa 0.46 mm long, 0.24 mm wide; metatrochanter 0.24 mm long, 0.10 mm wide; metafemur 1.02 mm long, 0.10 mm wide, with trochantellus and small, tapered setae at lower end; metatibia 0.92 mm long, 0.11 mm wide, thick pectinate metatibial spur paired with thinner simple spur of same length at terminal end. Each leg with minute trochantellus present. Tarsal segments setose, with four thick setae per segment at apex of each tarsomere. Pretarsal claws with subapical tooth; whorl of approximately 5 pairs of long setae near arolium.

**Metasoma**: Petiole nodiform, pedunculate, 0.40 mm total length (peduncle 0.09 mm); attaching to propodeum at a height of 0.13 mm in lateral view. Nodiform dorsal projection gradually rounded, approximately 0.30 mm high, reduced to 0.15 mm at helcium. Helcial sternite distinct and elongate with clear sulci, 0.10 mm length. Abdominal segments 3–7 appear slightly telescoped, not fully exposed. Gastral segment I (abd segment III) narrowed greatly and extended 0.12 mm anteriorly to meet petiole, possessing pointed sternal projection underneath this extension (0.04 mm long). Gastral segment I (incl extension) 0.42 mm long, segment II 0.57 mm, segment III 0.37 mm, segment IV 0.26 mm, segment V setose 0.22 mm. Sting extruded, 0.41 mm exposed.

Types: Holotype JZC Bu222A. Wingless female (presumed worker). Preserved in darkened orange-colored amber, trimmed to 11×6×4 mm. Amber also contains particles and detritus, plant trichomes. Curiously, the subapical tooth of one mandible appears to be broken off. Paratypes JZC Bu224, JZC Bu111, JZC Bu301, JZC Bu112, JZC Bu105-A, JZC Bu302-A.

Etymology: In description of distinctive spiraling frontal carina.

Comments: This species appears to be the most common in Burmese amber, comprising the greatest percentage (∼25%) of currently known and identifiable Burmese ant workers.

***Sphecomyrmodes subcuspis,*** **new species**

urn:lsid:zoobank.org:act:1AC94161-01E9-42CA-8BBF-3C837D433D3C 

Figures 8A–C.

**Figure 8:**
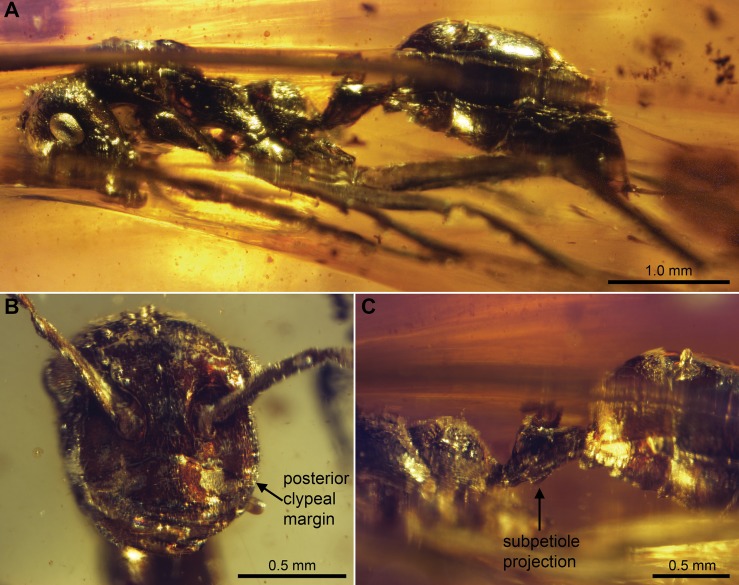
*Sphecomyrmodes subcuspis* holotype JZC Bu304 photomicrographs. A. Lateral view of entire body, partially obscured by fissure in amber. B. Frontal view of head. Lateral view of propodeum, petiole, and anterior sclerites of gaster.

Worker Diagnosis: Differentiated on basis of acute ventral petiole projection with sheer anterior face; clypeus setose, rounded posteriorly. Total length 5.35–5.76 mm.

Description: **Head**: Capsule somewhat square, in frontal view length from posterior margin of head to anterior margin of clypeus 0.95 mm, width just below eyes 0.93 mm. Width of face varies little, except for gradual rounding at dorsal margin. Head in profile somewhat drop-shaped, narrowed 40% from median margin of eyes to clypeus. Occipital carina oval, with smooth uninterrupted edges. Ocelli not observable [if present, obscured by particulate matter and bubbles]. Eye length 0.26 mm, width 0.19 mm, midline positioned approximately 70% toward occipital foramen from anterior margin of clypeus. Eyes bulging greatly in frontal view, inner margins of eyes separated 0.74 mm. Antennal socket exposed, torulus well developed, raised to partially cover antennal bases. Frontal carina sinuous, spiral from anterior margin of antennal socket to just lateral of bottom edge of eye. Cuticle raised between antennae. Total antenna length 3.34 mm; scape 0.62, pedicel 0.15, funicular segment I 0.49, II 0.35, III 0.32, IV 0.34, V 0.30, VI 0.24, VII 0.27, VIII 0.30, IX 0.32, ×0.41. Segments of roughly equal thickness throughout, devoid of macrosetae. Posterior margin of clypeus broadly notched, gradually joined with lateral margin at very wide (∼160°) angle. Clypeal sclerite raised along medial axis, with numerous setae of varied lengths (as long as 0.15 mm) on dorsal surface. Entire anterior margin of clypeus convex, with row ca. 30 slightly pointed denticles, approximately 0.03 mm in length, these denticles appear as two rows in some sections of clypeal margin. Anterolateral margin of clypeus raised as a slightly upturned shelf above base of mandible in frontal view. Mandibles bidentate and falcate, 0.69 mm long from base to tip of apical tooth; apical tooth 0.16 mm long, 0.06 mm wide at base, basal tooth measurements 0.08 mm, 0.09 mm. Labial palps comprised of two equal-sized palpomeres, maxillary palps not visible.

**Mesosoma**: Some features of mesonotum obscured by fracture through ant inclusion. Pronotum extended and rounded to accommodate and meet occipital foramen, which is rounded in dorsal view. Weber’s length 2.11 mm. Pronotum length 0.92 mm dorsally, mesonotum 0.43 mm, metanotum 0.32 mm, propodeum 0.65 mm. Pronotal-propleural sulci well developed, promesonotal suture appears very deep, metanotal groove also prominent. Propodeal spiracle situated at tip of cuticular projection near anterior margin of sclerite; projection oriented posterodorsad. Metapleural gland opening visible as indentation just above metacoxa (a small air bubble protruding). Legs extremely long, with very sparse setae. Procoxa 0.67 mm long, width obscured, protrochanter 0.23 mm long, 0.17 mm wide; profemur 1.41 mm long, 0.17 mm wide, trochantellus visible; protibia 1.11 mm long, 0.12 mm wide; protibial spur pectinate, with two accompanying setae attached to tibia approximately 0.3× length of spur. Mesocoxa 0.37 mm long, 0.24 mm wide at base, 0.14 mm at apex; mesotrochanter 0.23 mm long, 0.16 mm at widest; mesofemur 1.47 mm long, 0.14 mm wide, trochantellus present; mesotibia 1.36 mm long, 0.12 mm wide; two mesotibial spurs present, one pectinate, the other simple and slightly shorter, with single short, stiff seta at spur base. Metacoxa 0.65 mm long, 0.14 mm wide at base, 0.20 at apex; metatrochanter 0.26 mm long, 0.14 mm at widest point; metafemur 1.17 mm long, 0.09 mm wide, trochantellus visible; metatibia 1.78 mm long, 0.15 mm wide. Two metatibial spurs present, one thick and pectinate, one thin and simple. Tarsus setose along inner margin, terminus of each tarsomere with four prominent, pointed setae; distitarsomere with prominent arolium, six projecting setae, claw with subapical tooth.

**Metasoma**: Petiole nodiform, with shortened peduncle and rounded dorsal node. Propodeal-petiolar attachment height 0.16 mm. Petiole length 0.59 mm in lateral view (peduncle 0.11 mm.) Node gradually rounded, height 0.36 mm, shortened to 0.18 mm at gaster attachment point. Ventral projection of petiole present, originates approximately at center of petiole, gradually increased in height anteriad to 0.06 mm at its apex, with sheer anterior face. Gastral segment I (abdominal segment III) extended 0.10 mm anteriorly, meeting petiole with projection pointed ventrad (projection 0.08 mm long). Segment I length 0.55 mm, including anterior extension, gastral segment II length 0.60 mm, segment III 0.35 mm, IV 0.20 mm, V 0.29 mm. Pygidium setose; sting extruded, 0.95 mm exposed.

Types: Holotype JZC Bu304. Wingless female (presumed worker). Specimen preserved in 29×13×4 mm piece of very clear yellow amber. Also preserved are particles of detritus, bubbles, a partial spider, and an insect larva (not an ant). Paratypes JZC Bu305, JZC Bu121A.

Etymology: From cuspis, Latin for point, in reference to the petiolar projection; the prefix in reference to the ventral position of this point.

***Sphecomyrmodes tendir,*** **new species**

urn:lsid:zoobank.org:act:81A4DB69-1E5E-4418-9578-A212E796790F 

Figures 9A–C.

**Figure 9:**
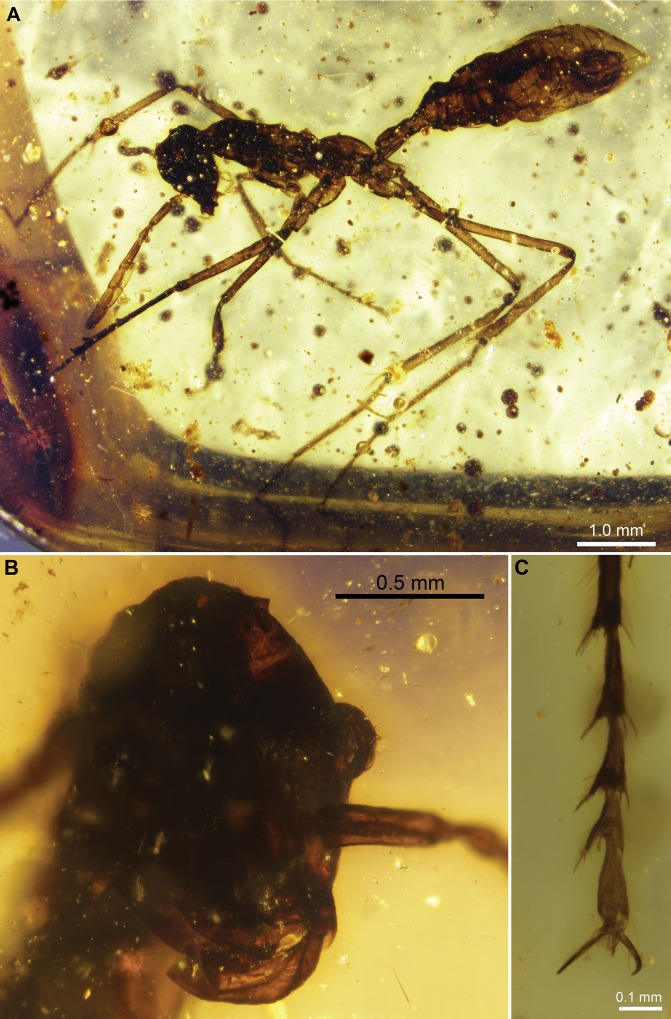
*Sphecomyrmodes tendir* holotype JZC Bu303A photomicrographs. A. Lateral view of entire specimen, other views not possible due to severe cuticle darkening. B. Anterolateral view of head. C. Enlarged view of tarsus demonstrating reduced subapical tooth on pretarsal claw.

Worker Diagnosis: Differs from congeners based on the clypeal setae, which do not cover the entire anterior margin as in other *Sphecomyrmodes*, but rather only a medial portion of the anterior margin where there is also a prominent medial lobe; a reduced subapical tooth on the pretarsal claw. Body length 6.93 mm.

Description: **Head**: elongate, even in width throughout. Height from anterior ridge of clypeus to posterior margin of head 1.22 mm, width greatest below eyes, 0.78 mm. Medial margin of eyes positioned ∼55% the distance from anterior margin of clypeus to posterior margin of head; eyes small, bulging when viewed head-on, 0.11 mm deep, 0.06 mm wide. Cuticle heavily darkened, fine sutures and details obscured, ocelli not visible. Two small, pointed lobes present on vertex of head. Cuticle raised between antennae, antennal bases exposed (measurement not possible due to darkening). Funicular segments IX and X missing. Scape 0.42 mm long, pedicel 0.16, funicular segment I 0.38, II 0.31, III 0.30, IV 0.20, V 0.25, VI 0.30, VII 0.27, VIII 0.25. Clypeus with medial anterior expansion originating 0.11 mm from mandible base, 0.10 mm deep at apex; anterior margin of this expansion with row of approximately 20 pointed setae (0.02 mm long). Anterolateral margin of clypeus with shelf-like, pointed anterior projection covering mandible bases. Mandibles glabrous, bidentate with apical tooth slightly larger than subapical. Maxillary palps with four, approximately equally-sized palpomeres; labial palps with two equal palpomeres.

**Mesosoma**: Entire mesosoma glabrous. Weber’s length 2.35 mm. Pronotum length 0.78 mm, mesonotum 0.55, metanotum 0.32, propodeum 0.74. Pronotum-head capsule attachment point obscured by darkened cuticle. Mesonotum with rugose sculpturing, metanotal spiracle opening protruding, surrounding cuticle rounded. Propodeal spiracle near anterior margin of sclerite, present on top of dorsal projection; metapleural gland opening very large (0.15 mm at greatest width) just above metacoxa. Propodeum gradually rounded posterodorsally, petiole attaches at posteroventral margin. Procoxa 0.69 mm long, 0.30 mm wide at base, 0.19 mm at apex; protrochanter 0.31 mm long, 0.16 mm wide; profemur 1.43 mm long, 0.18 mm wide with trochantellus present; protibia 0.74 mm long, 0.15 mm wide; single protarsal spur possesses small subapical tip, pectinate between this tip and apex. Mesocoxa 0.56 mm long, 0.33 mm wide at base, 0.24 mm wide at apex; mesotrochanter 0.32 mm long, 0.13 mm wide; mesofemur 1.44 mm long, 0.13 mm wide with trochantellus; mesotibia 1.34 mm long, 0.08 mm wide with numerous tapered setae toward apex; two mesotibial spurs of equal length present, one simple, one pectinate. Metacoxa 0.59 mm long, 0.24 mm wide at base, 0.17 mm wide at apex; metatrochanter 0.30 mm long, 0.15 mm wide; metafemur 1.60 mm long, 0.16 mm wide, slightly setose; metatibia 2.06 mm long, 0.18 mm wide, with tapered setae toward apex; one simple and one pectinate tibial spur of equal length. Tarsomeres terminate with four stiff, pointed setae; pretarsal claw with very slight, reduced subapical tooth.

**Metasoma**: Petiole cylindrical, with a slight peduncle, total length 0.70 mm (including peduncle [0.13 mm]) attached to propodeum at height of 0.10 mm in lateral view, increasing to 0.31 mm at apex; dorsally setose with long, tapered setae up to 0.13 mm in length; helcium a distinct sternite with a length of 0.16 mm, attaching to gastral segment I (abdominal segment III) at a height of 0.24 mm. Gaster elongate, desiccated and slightly distorted. Gastral segment I (abdominal segment III) 0.42 mm, II 0.43, III 0.41, IV 0.35, V 0.39. Pygidium with ring of small, tapered setae; sting extruded and visible through cleared gaster, 0.57 mm in total length.

Types: Holotype JZC Bu303A. Wingless female (presumed worker) preserved in 15×8×4 mm section of transparent yellow amber, surrounded by small particles of detritus. Paratype JZC Bu303B.

Etymology: From the latin word “tendo” meaning to extend. Referring to the medial clypeal projection.

Comments: The severely darkened cuticle of the specimen renders resolving some detailed characters impossible with light microscopy. The distinctive medial lobe on the anterior margin of the clypeus is similar to that of *Sphecomyrma mesaki* Engel and Grimaldi, however more broad.

#### Key to *Sphecomyrmodes* Workers

This key is intended for identification of *Sphecomyrmodes* workers presently known from Burmese and Charentese amber.

**Figure 10:**
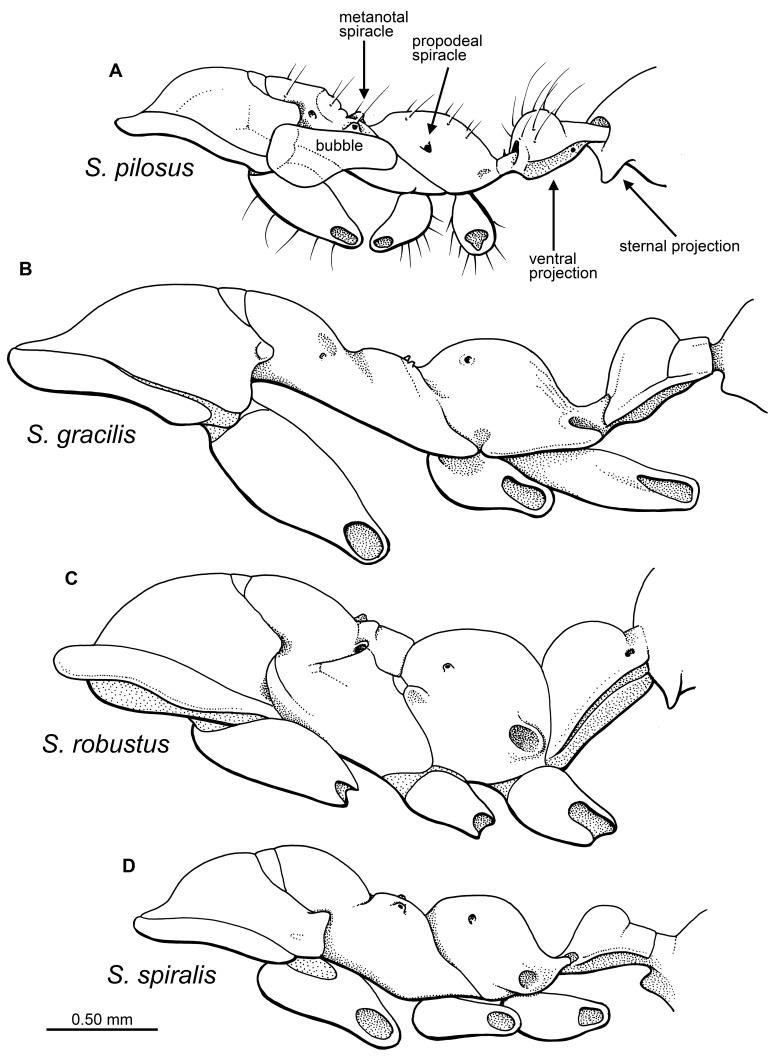
Drawings of *Sphecomyrmodes* mesosoma and partial metasoma in lateral view. A. *S. pilosus* holotype JZC Bu225. B. *S. gracilis* holotype JZC Bu324A. C. *S. robustus* holotype JZC Bu223A. D. *S. spiralis* holotype JZC Bu222A.

1. Anterior margin of clypeus with conspicuous medial lobe, setae present only along this lobe, not entire margin… ***tendir***
  Clypeus without such a lobe or if minor lobe present, setae span entire anterior margin…2
2. Petiole possesses a ventral projection with a flattened anterior face (as in figure 10A) …3
  Ventral projection gradually rounded anteriorly or no projection present (as in figures 10B,C)…4
3. Head, mesosoma, and metasoma heavily coated in tapered setae (as long as 0.25 mm); dorsal constriction between gastral segment I and II (abdominal segments III and IV). … ***pilosus***
  No such coating, surface of head and body nearly devoid of setae; no constriction between gastral segment I and II (abdominal segments III and IV)… ***subcuspis***
4. Antennal scrobe present, leading from antennal base to compound eye, scrobe approximately same length as scape… ***contegus***
  No scrobe present…5
5. Petiole sessile; dorsal surface of clypeus with numerous long (≥∼0.20 mm) tapered setae extending over mandibles…6
  Petiole pedunculate; dorsal clypeal surface glabrous or with arrangement of short setae …7
6. Total body length excluding sting and antennae ≥8.0 mm… ***magnus***
  Total body length excluding sting and antennae <6.0 mm… ***robustus***
7. Pointed sternal projection present on gaster segment I (abdominal segment III) (as in figures 10A–D)…8
  Ventral surface of gaster segment I with no projection…10
8. Head and body cuticle smooth, with no rugosity; maxillary palps with four palpomeres …9
  -Head and body cuticle rugose; maxillary palps with six palpomeres… ***rugosus***
9. Mesopleuron extremely elongate such that the distance between the pro- and mesocoxae is roughly twice the procoxal length… ***gracilis***
  Mesopleuron fairly stout, distance between pro- and mesocoxae equal to or less than width of procoxae… ***spiralis***
10. Setae present on external margins of mandibles… ***orientalis***
  External margins of mandibles entirely glabrous… ***occidentalis***

### Discussion

#### Comparison with Modern and Other Cretaceous Taxa

**Figure 11:**
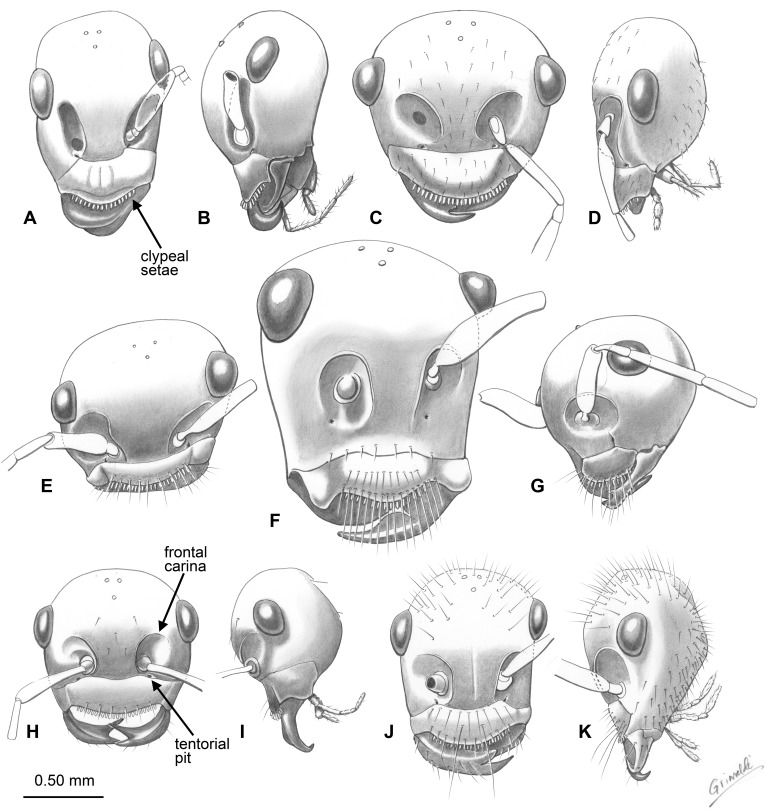
Drawings and partial reconstructions of *Sphecomyrmodes* heads. A. Frontal view of *S. contegus* holotype JZC Bu300A. B. Lateral view of *S. contegus* holotype. C. Reconstructed frontal view of *S. gracilis* holotype JZC Bu324A. D. Lateral view of *S. gracilis* holotype. E. Anterolateral view of *S. robustus* holotype JZC Bu223A. F. Frontal view of *S. magnus* holotype JZC Bu108A. G. Frontal view of *S. robustus* holotype. H. Reconstructed frontal view of *S. spiralis* holotype JZC Bu222A. I. Lateral view of *S. spiralis* holotype. J. Frontal view of *S. pilosus* holotype JZC Bu225. Lateral view of *S. pilosus* holotype.

**Figure 12:**
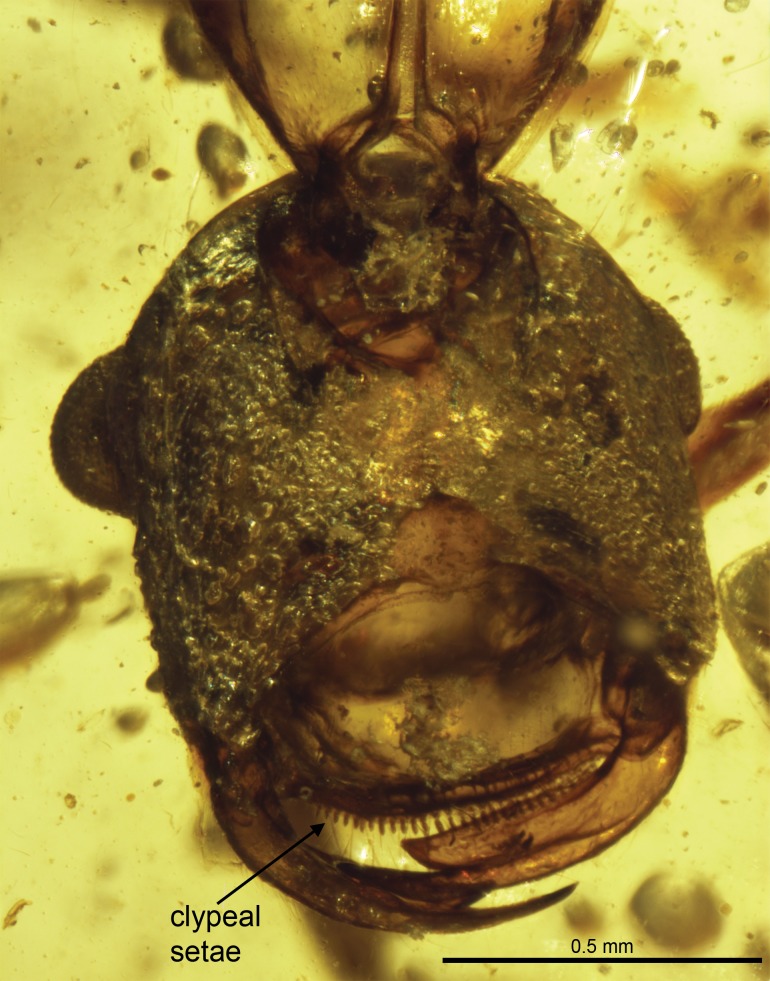
*Sphecomyrmodes spiralis* paratype JZC Bu301 photomicrograph. Ventral view of head. Paratype is partially disarticulated, however provides a clear view of anterior clypeus margin.

Each species described here exhibits a unique assemblage of synapomorphic ant features, bygone primitive morphology, and characters not before known in stem-group taxa (figures 10, 11). Moreover, all of these animals possess a clearly discernible shared character, suggesting surprising diversity among closely related species. Described species possess a metapleural gland opening, petiole, and geniculate antennae, as in modern ants. Unlike crown-group ants, these species possess a shortened scape, comprising ≤0.25× the total antennal length, a feature of stem-group ants [3] [18]. Members of the extinct genus *Sphecomyrmodes* also possess a comb of stout setae along the anterior clypeal margin (figure 12). A similar, although possibly homoplastic, condition is found in some modern ants, such as *Adetomyrma, Amblyopone*, *Martialis*, and *Stigmatomma* [19]–[21], and, aside from long trigger hairs found in trap-jaw ants, the function of clypeal setae is unknown. A comb of clypeal setae is also known from the Cretaceous genera *Gerontoformica* and *Zigrasimecia* [11] [22]. *Zigrasimecia* possesses an additional set of setae on the labrum, and *Gerontoformica*, while similar in many respects to *Sphecomyrmodes*, can be distinguished by an elongated scape, a character that may have importance with regard to the crown- or stem-group affinities. Despite the diagnosable differences among clypeal setae bearing ants, it seems likely that these groups share close relationships.

The genus *Sphecomyrmodes* is readily placed in the putative stem-group subfamily Sphecomyrminae. However, new Cretaceous diversity along with other incertae sedis taxa such as *Myanmyrma* [6] and *Gerontoformica* [22] highlight the need for a rigorous phylogenetic analysis of Cretaceous taxa and stable classification. Some group features, such as the gastral constriction present in *S. pilosus* (figure 4a), indeed appear to vary within stem-group ant lineages.

#### Unexpected Diversity

With a total of 11 species – nine described here, two described previously [6]–[7] – the genus *Sphecomyrmodes* represents significant diversification among stem-group ants united by a distinct trait. It should also be noted that the discovery of two *Sphecomyrmodes* workers in slightly older French amber [7] indicate that the genus had considerable geographic range. Anterior clypeal setae appear to have been useful for these early species, as they remain a consistent feature among a varying stock of morphological diversity (figures 10, 11, 12). Although these individuals have been placed within the same genus some of the features that vary among them are used in present-day taxa to separate higher-level groups. *Sphecomyrmodes* species are now known to vary with regard to scrobe presence, frontal carinae structure, petiole form, and palpomere count. There is also considerable diversity among comparable species-level traits such as pilosity, fine cuticular sculpturing, and body form. The range of overall body size and proportionality is quite large, with the smallest specimen of *S. spiralis* at just 4.22 mm in length, contrasted with the largest individual *S. magnus* at a total length of 8.64 mm. Mesosomal elongation varies as well, with a ratio value (length/height) twice as high in *S. gracilis* as *S. magnus*. In terms of gaster length, proportionally, the fully extended gaster of *S. contegus* comprises just 25% of total body length, while the gastral segments of *S. gracilis* make up 50% of total body size.

Ancient ant stem-groups were not morphologically stagnant. Indeed, ant species from the Cretaceous exhibited species-level radiations comparable to modern day taxa with similar variations of common features. This apparent homoplasy may be highly informative in understanding patterns of ant evolution as we now know that stem-group (that is, of Cretaceous age not possessing an elongated scape) ants appear to have the same innovations that their modern counterparts exhibit such as: feeding morphologies (mandibular, palp structure, setae patterning), head sculpturing (elongate, equilateral, widened), body form (gracile, robust, sculptured, setose), fine sculpturing (rugosity, pilosity, spiracle placement, sub-petiole and gastral projections), leg morphology (claw morphology, setae patterns). There is no clear single “linking-ant,” instead it is apparent that there was significant Cretaceous diversity and innovation, in this case curiously united by a clypeal peg structure that is known in extant basal lineages.
